# Genotype-specific germination behavior induced by sustainable priming techniques in response to water deprivation stress in rice

**DOI:** 10.3389/fpls.2024.1344383

**Published:** 2024-02-08

**Authors:** Conrado Dueñas, Andrea Pagano, Cinzia Calvio, Dhanush Srikanth Srikanthan, Inez Slamet-Loedin, Alma Balestrazzi, Anca Macovei

**Affiliations:** ^1^ Department of Biology and Biotechnology ‘L. Spallanzani’, University of Pavia, Pavia, Italy; ^2^ Trait and Genome Engineering Cluster, Rice Breeding Innovations, International Rice Research Institute, Metro Manila, Philippines

**Keywords:** poly-gamma glutamic acid (γ-PGA), polyethylene glycol (PEG), iron pulsing, seed germination, gene expression, antioxidant response, DNA damage response

## Abstract

Water stress brought about by climate change is among the major global concerns threatening food security. Rice is an important staple food which requires high water resources. Being a semi-aquatic plant, rice is particularly susceptible to drought. The aim of this work was to develop techniques directed to promote rice resilience to water deprivation stress during germination by implementing specific seed priming treatments. Five popular Italian rice varieties were subjected to priming treatments using novel, sustainable solutions, like poly-gamma-glutamic acid (γ-PGA), denatured γ-PGA (dPGA), and iron (Fe) pulsing, alone or in combination. The effect of the developed priming methods was tested under optimal conditions as well as under water deprivation stress imposed by polyethylene glycol (PEG) treatments. The priming efficacy was phenotypically determined in terms of germination behavior by measuring a series of parameters (germinability, germination index, mean germination time, seed vigor index, root and shoot length, germination stress tolerance index). Biochemical analyses were carried out to measure the levels of iron uptake and accumulation of reactive oxygen species (ROS). Integrative data analyses revealed that the rice varieties exhibited a strong genotype- and treatment-specific germination behavior. PEG strongly inhibited germination while most of the priming treatments were able to rescue it in all varieties tested except for Unico, which can be defined as highly stress sensitive. Molecular events (DNA repair, antioxidant response, iron homeostasis) associated with the transition from seed to seedling were monitored in terms of changes in gene expression profiles in two varieties sensitive to water deprivation stress with different responses to priming. The investigated genes appeared to be differentially expressed in a genotype-, priming treatment-, stress- and stage-dependent manner. The proposed seed priming treatments can be envisioned as sustainable and versatile agricultural practices that could help in addressing the impact of climate challenges on the agri-food system.

## 1 Introduction

Re-emergence of global hunger is related to increasing obstacles in agricultural food production associated with the climate change scenario (https://www.fao.org/publications/sofi/2022/en/). Actions to mitigate these challenges are envisioned in the Sustainable Development Goals (SDG) Agenda 2030, mainly in SDG#2 - Zero hunger. Rice has high potential for aiding the accomplishment of SDG#2 due to its global consumption. This staple crop contributes to 35 - 59% of the daily caloric intake for billions of people, being the third most cultivated crop in the world (Sperotto et al., 2012). As a semiaquatic plant, rice is particularly sensitive to drought, a condition progressively worsening in the current climate scenario (Shahzad et al., 2021; Habib-Ur-Rahman et al., 2022). Hence, there is a need to develop strategies that could mitigate drought stress, starting from the germination stage (Panda et al., 2021).

Seed germination is one of the most important processes in the life of the plant, marking the transition from the quiescent state to a metabolically active state, initiating a series of biophysical, biochemical and molecular changes leading to radicle emergence and subsequent seedling growth (Han and Yang, 2015; Macovei et al., 2017; Yu et al., 2021). Within the agricultural context, optimal seed germination ensures seedling vigor and subsequently high yields during harvest, which are however negatively influenced by water deprivation stress (Osakabe et al., 2014; Reed et al., 2022). In the case of rice, numerous drought stress-related studies have been conducted at the reproductive stage due to its direct relation to yield (Rang et al., 2011; He and Serraj, 2012; Favreau et al., 2023; Lou et al., 2023) while less attention has been given to the germination stage.

Mature seeds contain an embryo in a quiescent state, characterized by reduced metabolism, although transcriptional and post-transcriptional modifications in response to the environmental signals remain partially active to sense and start the germination process under favorable conditions (Holdsworth et al., 2008). Seed germination is a triphasic process, where phase I is characterized by rapid water uptake, which in rice, corresponds to the first 20 h of imbibition. Phase II (lag phase) spans from 20 to 48 h, whereas phase III, representative of radicle emergence, starts after 48 h from imbibition (Yang et al., 2007). Complex physiological and biochemical events are involved in the transition from the dry, quiescent seed to the metabolically active state, including an intermediary state called pre-germinative metabolism, characterized by highly dynamic changes in carbohydrate metabolism, signal transduction, DNA synthesis, gene expression, regulation of redox homeostasis, and DNA repair (Howell et al., 2009; Macovei et al., 2017; Sano et al., 2022). Moreover, the *de novo* transcription happening during the early stages of germination is further subjected to additional regulation during seedling establishment (Rajjou et al., 2012; Sano et al., 2012). During the reactivation of the seed metabolism, reactive oxygen species (ROS) are produced at high levels due to rapid water uptake, and this can be potentially harmful if their levels pass a certain threshold (Baily et al., 2008, Pagano et al., 2022). To maintain ROS balance, the activation of antioxidant defense mechanisms are required to ensure successful seedling development (Kranner et al., 2010; Rajjou et al., 2012; Pagano et al., 2023). Moreover, efficient activation of the DNA damage response (DDR) pathway is necessary to repair DNA damage and safeguard seedling genome integrity (Balestrazzi et al., 2011; Pagano et al., 2017; Waterworth et al., 2019).

Generally, water deprivation stress can occur when water availability is low, potentially triggering loss of turgor pressure at a cellular level. In an agronomic context, water deprivation stress represents only a part of the complex drought conditions which highly restricts plant growth by reducing cellular elongation and expansion, detrimentally affecting seedling development and germination (Bhandari et al., 2023). Rice is highly sensitive to dry conditions during germination and the early phases of seedling growth. Low water potential in the soil prevents seedling from absorbing water, reducing seedling vigor (Kruthika and Jithesh, 2023; Székely et al., 2023). The effects of water deprivation stress include altered membrane transport, disruption of metabolic processes, decreased ATP synthesis, leading to aborted germination, or severe seedling growth reduction (Batlang et al., 2013; Oladosu et al., 2019; Kota et al., 2023). Transcriptional and hormonal activities, along with ROS levels, are highly affected when imbibition and seedling development occur during water stress conditions (Ishibashi et al., 2018; Kawaguchi et al., 2023).

Water deprivation stress at the germination stage can be addressed through several practices that include seed treatments to induce osmoprotection, limiting ROS accumulation, or enhancing the seed repair response. Among these treatments, seed priming is envisaged as a sustainable, cost-effective, and easy to use technique dedicated to improve seed germination even under adverse conditions. Seed priming is a pre-sowing technique that involves controlled imbibition, allowing seeds to reach the early stages of germination without radicle protrusion, followed by subsequent drying (Paparella et al., 2015; Devika et al., 2021). These treatments allow increased and uniform germination by acting on enhancing enzyme activation, metabolite production, repair of damaged DNA and osmotic regulation (Gerna et al., 2018; Forti et al., 2020a; Forti et al., 2020b; Wang et al., 2022). Even upon exposure to drought stress, this technique can speed up seedling emergence through the regulation of metabolic process in the early phases of germination (Marthandan et al., 2020). In rice, different priming methods, including hydropriming (Nakao et al., 2020), nanopriming (Waqas Mazhar et al., 2022), chemical priming applied through the use of salt solutions (Ali et al., 2021), plant extracts (Khan et al., 2022) or plant growth elicitors (Samota et al., 2017), have been applied and were shown to mitigate the effects of drought stress at different levels. Although the research in this field is steadily advancing, there is still the need to understand how priming works at a molecular level, and develop strategies that could mitigate water deprivation stress starting from the germination stage, taking into consideration also the intraspecific genetic variability (Pagano et al., 2023).

The priming protocols developed in this study utilize novel seed priming agents, namely, poly-gamma-glutamic acid (γ-PGA), denatured γ-PGA (dPGA), and iron (Fe) pulsing, applied to five economically important Italian rice varieties. Polyethylene glycol (PEG) was used to simulate water deprivation stress, as this is an inert and water-binding polymer with a non-ionic and impermeable long chain which mimics water and osmotic stress (Perez-Fernandez et al., 2006). γ-PGA, used as a chemical priming agent, has the potential to alleviate the effect of water stress due to its hygroscopic and water-holding qualities (Ogunleye et al., 2015). Additionally, dPGA can be used as a nutripriming agent given that it is constituted of single glutamic acid molecules released upon γ-PGA degradation. The combination of Fe pulsing with either γ-PGA or dPGA was also conducted to investigate a possible cumulative effect on rice germination under water deprivation stress. Phenotyping (germination, seedling growth, and stress parameters), biochemical (ROS detection, Fe quantification), and molecular analyses (gene expression profiling), were carried out to investigate the effect of priming and stress conditions on rice germination.

## 2 Materials and methods

### 2.1 Seed materials and water stress treatments

A selection of five popular Italian rice (*Oryza sativa* L.) varieties, namely Carnaroli, Cerere, Unico, Lomello, and Apollo, were used for the current study. Seeds were kindly supplied by Sardo Piemontese Sementi (Sa.Pi.Se, https://sapise.it/) and the University of Pavia Botanical Garden Plant Germplasm Bank (https://terraeambiente.dip.unipv.it/it/dipartimento/risorse/banca-del-germoplasma-vegetale).

To impose water deprivation stress during seed germination, a 25% PEG6000 (polyethylene glycol) solution was used to simulate an osmotic potential of -1215.9 kPa (Stanley and Strey, 2003) while distilled water (0 kPa) was used as a non-stress control. Before selecting this concentration, preliminary trials using 15%, 20% and 25% PEG were carried out on the Lomello variety. Petri dishes, containing 30 seeds/replicate, lined with a two-layer filter paper and 7 ml of PEG6000 or water, were placed in a growth chamber at 26°C under dark for two days and subsequently at 16 h light/8 h dark cycle for 10 days. The light conditions included a photon flux density of 150 μmol m^-2^s^-1^ and 70–80% relative humidity inside the containers. The system was laid out in a complete randomized design with three replicates.

### 2.2 Extraction of poly-gamma glutamic acid and denatured PGA

Recovery of crude γ-PGA was conducted by adapting a protocol from Massaiu et al. (2019). *Bacillus subtilis* strain PB5390 (Ermoli et al., 2021) was streaked on an LB plate and incubated at 37° overnight for single colonies. A pre-culture was prepared by incubating 6 colonies in LB liquid media containing 0.5% glucose at 37° with 233 rpm shaking for 8 h. The OD was measured at 600 nm to calculate the volume of the pre-culture that was added to the E-medium (40 g L^-1^ KC_5_H_8_NO_4_, 12 g L^-1^ Na_3_C_6_H_5_O_7_, 80 g L^-1^ glucose, 0.104 g L^-1^ MnSO_4_, 0.5 g L^-1^ MgSO_4_, 7 g L^-1^ K_2_HPO_4_, 0.04 g L^-1^ CaCl, 0.004 g L^-1^ FeCl, 0.05 g L^-1^ tryptophan, 0.05 g L^-1^ phenylalanine). The culture was incubated in the same conditions for 60 h. The culture was centrifuged (8,000 rpm, 15 min, 10°C) and the supernatant was collected. γ-PGA was precipitated with three volumes of cold methanol and kept at -20°C for 16 h. The mixture was later centrifuged to recover the γ-PGA pellet. The pellet was resuspended in 100 mM NaCl. The concentration of isolated crude γ-PGA was measured using a spectrophotometer at 216 nm in a quartz cuvette. A standard calibration curve with commercial γ-PGA was used to compute its concentration in g L^-1^. Absorbance measurement was carried out via the NanoPhotometer UV/Vis spectrophotometer (Implen, Schatzbogen, München, Germany), using 100 mM NaCl as blank. Denatured γ-PGA (dPGA) was prepared by incubating the extracted γ-PGA in a 120° oven for 72 h.

### 2.3 Priming treatments

For γ-PGA and dPGA priming treatments, sets of 30 rice seeds were placed in an open mouth flat bottomed tube and filled with 3 ml of 1 g L^-1^ γ-PGA or dPGA. Floating seeds on the surface were gently tapped to ensure total submergence in the soaking solution. The seeds were soaked for 16 hours at room temperature before direct sowing in Petri plates containing 25%PEG solution or water.

The iron (Fe) pulsing protocol used in this study was adapted from Dey et al. (2021). A volume of 3 ml of water was poured into an open mouth-flat bottom tube containing 30 rice seeds per replicate. The seeds were soaked for 16 hours at room temperature. Afterward, the seeds were sown directly on 85 mm plastic Petri dishes lined with 2 filter papers soaked in 7 ml of 10 mM ferrous sulphate heptahydrate (FeSO_4_.7H_2_0). Alternatively, iron pulsing was combined with either γ-PGA (treatment PGA-Fe) or with dPGA (treatment dPGA-Fe) priming. For the PGA-Fe and dPGA-Fe priming, water was replaced with a soaking solution of either 0.001 g L^-1^ γ-PGA or 0.001 g L^-1^ dPGA. The sowing for Fe treatment started after soaking from Day 0 of the experiment. After 3 days, the seeds were transferred to new Petri plates with 2 filter papers and 7 ml of distilled water (non-stressed condition) or 25%PEG (stressed condition) and allowed to grow for a duration of 10 days.

Water soaking (WS) was included among the treatments to serve as a control for distinguish between the effect of the different agents used (PGA, dPGA, Fe) from that of water. Moreover, WS can also be considered as a priming treatment and it was previously used as a traditional pre-sowing technique in rice cultivation (Farooq et al., 2006). For the WS treatment, rice seeds were soaked in water for 16 hours at room temperature and subsequently directly sown in the 25%PEG- or water-containing Petri dishes.

**Figure 1:**
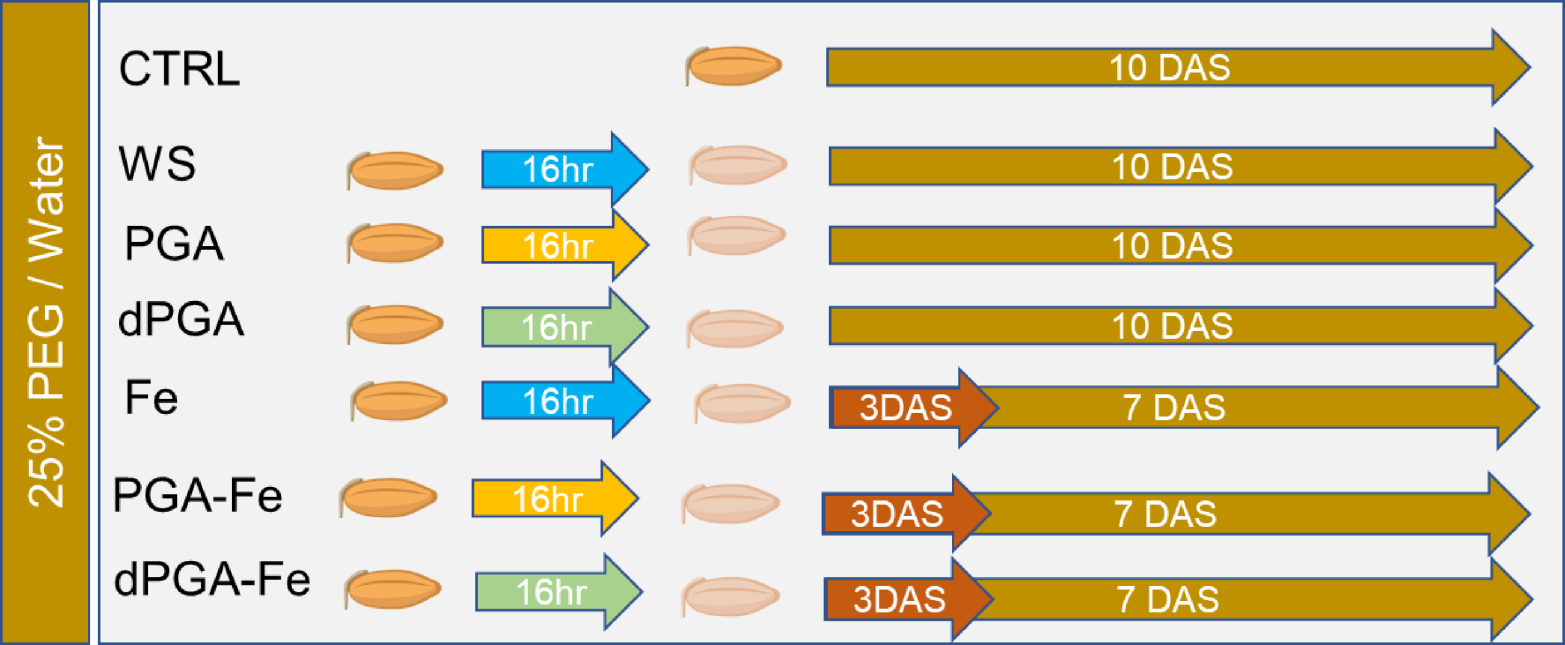
Overview of the experimental system developed to compare the effects of proposed seed priming protocols on the germination behavior of Italian rice varieties under 25%PEG-induced stress or non-stress (water) conditions. CTRL, unprimed control; WS, water soaking; PGA, poly-gamma-glutamic acid; dPGA, denatured poly-gamma-glutamic acid; Fe, iron pulsing; DAS, days after sowing. Blue arrow indicates that seeds were soaked for 16 h in water. Yellow arrow indicates that seeds were soaked for 16 h in PGA. Green arrow indicates that seeds were soaked for 16 h in dPGA.

A schematic representation of the experimental design based on different priming protocols and germination in the presence of 25%PEG-induced stress or non-stress conditions is given in **Figure 1**.

### 2.4 Phenotyping analyses

The number of germinated seeds was recorded at 24 h intervals. Seeds were considered as germinated when they reached the S2 stage, when both the plumule and radicle were 2 mm long (Counce et al., 2000). Different sets of parameters were collected and calculated to evaluate germination performance, seedling growth, and stress tolerance (Kader, 2005; Ranal and Santana, 2006; Mahmood et al., 2022). For the evaluation of germination performance, the following parameters were used: germinability (G%), germination index (GI), mean germination time (MGT), and synchronization index (Z). To assess seedling growth, root length (Root), shoot length (Shoot), and seedling vigor index I (VII), were used. To test the water deprivation stress resilience or sensitivity, several tolerance indices were selected as follows: germination water stress tolerance index calculated at 7 days (GDTIMax), shoot length water stress tolerance index (SLWTI), and root length water stress tolerance index (RLWTI). The formulas used for the calculations of each parameter and the explanation of the obtained values are given in **Supplementary Table 1**. Measurements of shoot and root length were taken using the ImageJ program (https://imagej.nih.gov/ij/download.html).

### 2.5 Histochemical staining for Fe and H_2_O_2_ detection

The presence of Fe in the embryo axis was evaluated using Perl’s Prussian staining protocol adapted from Shobhana et al. (2013). Seedlings were removed from the plates after 10 days from sowing, thoroughly washed with water, and soaked in Perl’s stain containing 2% potassium ferrocyanide (K_4_[Fe(CN)_6_]) and 2% hydrochloric acid (HCl), for 30 min at room temperature under dark and vacuum conditions. Blue color develops in the presence of Fe in the tissue.

The levels of hydrogen peroxide (H_2_O_2_) in the embryo axis tissue were evaluated using the 3,3’-diaminobenzidine (DAB) staining method (Macovei et al., 2010). The DAB solution was prepared at a 0.005 g L^-1^ concentration by dissolving 3,3’-diaminobenzidine tetrahydrochloride in Tris-Buffered Saline (TBS, 50 mM Tris-Cl, pH 7.5, 150 mM NaCl) buffer. At the end of the treatments, 10-day old seedlings were soaked in 0.005 g L^-1^ DAB stain for 1 h under dark and vacuum conditions. Brown color develops in the presence of H_2_O_2_ in the tissue.

For both types of histochemical staining methods, a semi-quantitative approach was carried out to estimate the presence of Fe or H_2_O_2_ in the embryo axis, and data are presented as fold change to control. The intensity color gradient was measured using the ImageJ program.

### 2.6 RNA extraction and cDNA synthesis

Samples from the PEG-induced stress were collected at 3 time points, namely 16 h, 3 and 7 days after sowing (DAS), along with the embryo axis extracted from dry (untreated) mature seeds. Relatively 80 to 90 mg of tissue for embryo axis and 100 to 150 mg of tissue for seedlings were grinded in liquid N and collected into 1.5 ml tubes. For RNA isolation the TRIzol extraction method was applied following manufacturer’s indications. A volume of 750 μl of TRIzol reagent (Thermo Fisher Scientific, Monza, Italy) was added to each tube, vortexed for 5 s, and incubated in ice for 5 min. A volume of 150 μl chloroform was added, gently mixed, incubated for 3 min on ice, and centrifuged at 10,000 rpm for 15 min at 4°C. The upper phase was collected and transferred to new 1.5 ml tubes and an equal volume of isopropanol was added. The samples were incubated at -20°C for 20 min then centrifuged with the same settings and the supernatant was discarded to collect the RNA pellet. The pellet was washed with 500 μl ice-cold 70% ethanol and centrifuged, followed by two additional washes with 500 μl ice cold absolute ethanol. Finally, the pellet was air dried and resuspended in nuclease-free water. The concentration of each RNA sample was measured using a Biowave spectrophotometer (Biochrom Ltd., England), and the integrity of RNA samples was assessed on agarose gel. For cDNAs synthesis, the RevertAid First Strand cDNA Synthesis Kit (ThermoFisher Scientific, Monza, Italy) was used, according to the manufacturer’s recommendations.

### 2.7 Quantitative real-time PCR

The qRT-PCR reactions were carried out on a CFX Duet Real-time PCR system machine (Bio-rad Laboratories Inc., Milan, Italy) using the Maxima SYBR Green qPCR Master Mix (Thermo Fisher Scientific, Monza, Italy) as indicated by the manufacturer. The machine was operated using the Bio-rad CFX maestro software (Bio-rad Laboratories Inc.) and the following amplification conditions were applied: denaturation at 95°C, 10 min, and 40 cycles of 95°C, 15 s and 60°C, 30 s, final extension at 72°C, 30 s.

Oligonucleotide primers (**Supplementary Table 2**) were designed using Primer3plus (https://www.primer3plus.com/index.html) and subsequently verified with Oligo Analyzer (https://eu.idtdna.com/calc/analyzer), and Primer-Blast (https://www.ncbi.nlm.nih.gov/tools/primer-blast/index.cgi?GROUP_TARGET=on). The list of investigated genes include: *LigVI (DNA LIGASE VI), FPG (FORMAMIDOPYRIMIDINE-DNA GLYCOSYLASE), NBS1 (NIJMEGEN BREAKAGE SYNDROME 1 MUTATED GENE), OGG1 (8-OXOGUANINE DNA GLYCOSYLASE), OPT7 (OLIGOPEPTIDE TRANSPORTER 7), PARP1 (POLY [ADP-RIBOSE] POLYMERASE 1), RAD51 (RADIATION SENSITIVE PROTEIN 51), TDP1α (TYROSYL-DNA PHOSPHODIESTERASE 1α), TDP1ß (TYROSYL-DNA PHOSPHODIESTERASE 1β), AoX1b (ALTERNATIVE OXIDASE 1B), APX6 (ASCORBATE PEROXIDASE 6), Cu/ZnSOD (CU/ZN SUPEROXID DISMUTASE), SODFe (FE SUPEROXID DISMUTASE), MIR (MITOCHONDRIAL-IRON REGULATED), NAS1 (NICOTIANAMINE SYNTHASE 1), NRAMP1 (NATURAL RESISTANCE-ASSOCIATED PROTEIN 1), VIT1 (VACUOLAR IRON TRANSPORTER), AAP1 (ASPARTATE AMINOTRANSFERASE 1), C3H50 (CYSTEINE 2 HISTIDINE)*. The *25sRNA* was selected as a reference gene given that it was previously reported to be stable across different growth conditions and developmental stages in rice (Jain et al., 2006). The Thomsen method was used for relative quantification of transcript accumulation using the efficiency value of 1.8 (Thomsen et al., 2010). All reactions were carried out in triplicates. For data representation, Z-score was calculated on the linearized Ct values and used to generate heatmaps using the OriginPro 2023b software (OriginLab Corporation, Northampton, MA, USA).

### 2.8 Statistical analyses

Statistical analyses were carried out using the Statistical Tool for Agricultural Research 2.0.1 (STAR 2.0.1) software. Significant differences among varieties and treatments were determined though a two-way ANOVA (Analysis of Variance) method. The significance of the results was evaluated at a 0.05% level (*p* ≤ 0.05) using the Least Significant Difference (LSD) or Tukey’s Honest Significant Difference (HSD) tests. Means with the same designated letters are indicated as not significantly different. For the qRT-PCR data, the Student *t*-test (*, p ≤ 0.05) was used for statistical interpretation where each priming treatment was compared to the non-primed control (CTRL). A Principal Component Analysis (PCA) was conducted using the Originpro 2023b software (https://www.originlab.com/2023), following user’s guide specifications.

## 3 Results

### 3.1 Seed priming mitigates the negative effect of water stress during germination

Seed of Apollo, Carnaroli, Cerere, Lomello, and Unico varieties were treated with either water (WS), PGA, dPGA, Fe, PGA-Fe-, and dPGA-Fe. An unprimed control (CTRL) was also used. Primed and unprimed seeds were sown in Petri dishes under control conditions and PEG-induced stress. To evaluate the effect of the priming protocols, several germination parameters were analyzed.

In the absence of stress, no significant changes were observed in terms of germination percentage between the priming treatments and unprimed seeds (**Supplementary Figure 1**). The WS treatment showed a significant effect on the germination of all the tested varieties, specifically in terms of GI (**Supplementary Table 3**). A genotype-dependent effect of priming was observed. In Apollo, WS and PGA-Fe treatments decreased the germination time (MGT) by a day while the root length of seedlings from WS-, PGA-Fe-, and dPGA-Fe-treated seeds were significantly longer than the CTRL. The PGA, PGA-Fe, and dPGA-Fe treatments also resulted in high vigor (VII) values. For Carnaroli, increased shoot growth was observed in seedlings from WS- and dPGA-Fe-treated seeds while VII was improved only after WS treatment. The Cerere variety showed enhanced root and shoot length following WS, PGA, and dPGA treatments. The MGT decreased by 3 days in the Lomello variety, leading to a significant increase in root length following WS application. Treatments with PGA-Fe and dPGA-Fe also increased root length in Lomello. Unico showed no change in germination parameters across all priming methods applied, except for the GI values that were slightly improved upon WS treatments. Germinability (Gmax) and synchronicity (Z) did not significantly vary throughout the priming treatments in all varieties.

To simulate water deprivation stress, a PEG concentration of 25%, corresponding to -1215.9 kPa (Stanley and Strey, 2003), reported as the maximal limit in previous studies in rice (Islam et al., 2018; Sobahan et al., 2022) was used in this study. A preliminary test, conducted on the Lomello variety (**Supplementary Figure 2**) using 15%, 20%, and 25% PEG, indicated that 25% PEG allowed a better distinction between primed and unprimed seeds. Therefore, this concentration was chosen for subsequent analyses. Among the tested varieties, Lomello was identified as water deprivation stress tolerant as it showed germination values of 32.33 ± 5.03% under simulated drought, while Apollo, Carnaroli, Cerere, and Unico were classified as stress-sensitive since they did not germinate under these conditions (**Supplementary Table 4**, CTRL Gmax). However, the germinability of Apollo and Cerere seeds was rescued by iron pulsing (Fe) alone or in combination with PGA (PGA-Fe) and dPGA (dPGA-Fe). Significantly improved Gmax, GI, and VII values were registered following these priming techniques under PEG stress. The significant difference in MGT observed for Carnaroli and Cerere may be attributed to the events taking place during the first phases of germination, started during the 16 h soaking treatments applied before stress induction, while the CTRL was represented by dry seeds. The Unico variety can be considered as highly susceptible to water deprivation stress since even after the priming treatments seeds it did not germinate in the presence of PEG.

**Figure 2:**
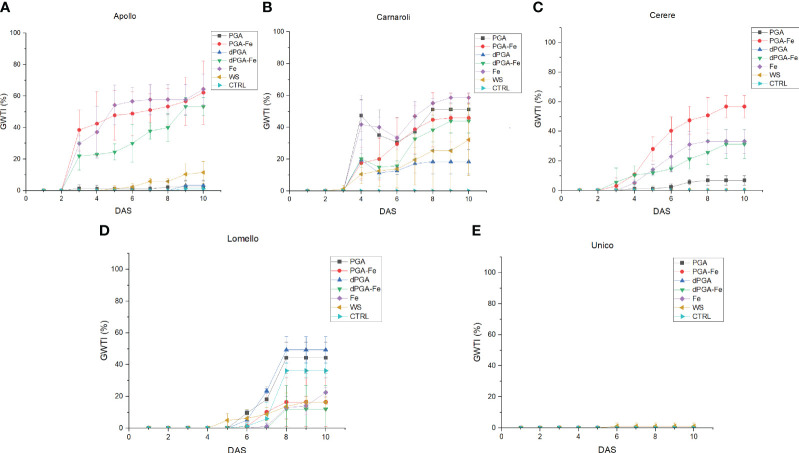
Germination water stress tolerance index (GWTI) for **(A)** Apollo, **(B)** Carnaroli, **(C)** Cerere, **(D)** Lomello, and **(E)** Unico. Data represent the means ± st.dev. of three independent replicates of 30 seeds each. CTRL, unprimed control; PGA, poly-gamma-glutamic acid; dPGA, denatured poly-gamma-glutamic acid; Fe, iron pulsing; WS, water soaking; DAS, days after sowing. Statistical analysis is provided in **Supplementary Table 5**.

Since there was an increase in germinability among the stress-sensitive varieties upon priming, the germination water stress tolerance index (GWTI), root length, shoot length, and seedling vigor (VII) were assessed. **Figure 2** shows the graphical progression of calculated values for each variety, while the statistical significance is provided in **Supplementary Table 5.** Overall, the previously observed trend was maintained among varieties also for these parameters. In terms of GWTI, Apollo showed a significant enhancement after treatments when Fe was applied either alone or in combination with PGA (PGA-Fe) and dPGA (dPGA-Fe) (**Figure 2A**). Carnaroli showed improved GWTI for most treatments (except dPGA) when compared to the CTRL conditions, and this was particularly significant for Fe pulsed seeds, reaching 58% at 10 DAS (**Figure 2B**). Cerere achieved elevated GWTI values up to 57%, specifically for the PGA-Fe-treated seeds (**Figure 2C**). For the highly-tolerant Lomello variety (**Figure 2D**) and the highly-sensitive Unico variety (**Figure 2E**), no significant changes were observed in terms of calculated GWTI values.

**Figure 3:**
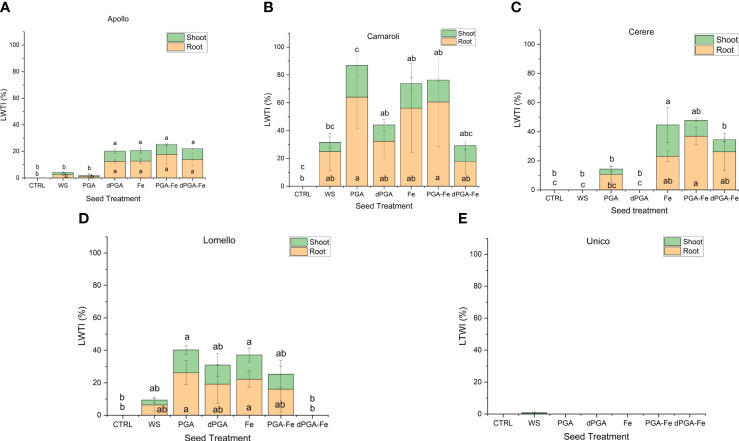
Calculated vigor tolerance indexes under stress in terms of shoot and root length (LTWI, length tolerance water stress index) for **(A)** Apollo, **(B)** Carnaroli, **(C)** Cerere, **(D)** Lomello, and **(E)** Unico. Data represent the means ± st.dev. of three independent replicates of 30 seeds each. CTRL, unprimed control; PGA, poly-gamma-glutamic acid; dPGA, denatured poly-gamma-glutamic acid; Fe, iron pulsing; WS, water soaking; DAS, days after sowing. Samples followed by different letters indicate statistically significant (*p* ≤ 0.05) differences determined using the ANOVA Tukey’s test.

When considering the calculated stress vigor indexes (**Figure 3**) similar patterns were observed among varieties. The elevated root length upon PEG-induced stress indicates that all seed priming treatments (except WS) applied on Carnaroli (**Figure 3B**), and iron pulsing (Fe, PGA-Fe, and dPGA-Fe) applied on Cerere (**Figure 3C**) and Apollo (**Figure 3A**), were able to mitigate the negative effect of water stress. The stress-tolerant Lomello variety showed significant differences compared to the CTRL for PGA and Fe pulsing treatments (**Figure 3D**), while no growth was registered in the case of Unico (**Figure 3E**) variety.

Overall, the proposed seed priming treatments were able to improve rice germination under both stress and non-stress conditions in a genotype-specific manner. The 25% PEG-induced stress strongly inhibited germination while most of the priming treatments were able to rescue it in all varieties tested except for Unico, which can be defined as highly sensitive to water deprivation stress. Importantly, the identification of Lomello as being highly stress tolerant and responsive to priming may help in enhancing its adoption not only for cultivation but also in breeding programs to further promote this trait.

### 3.2 The genotype-specific responses to priming are supported by PCA integrative analyses

**Figure 4:**
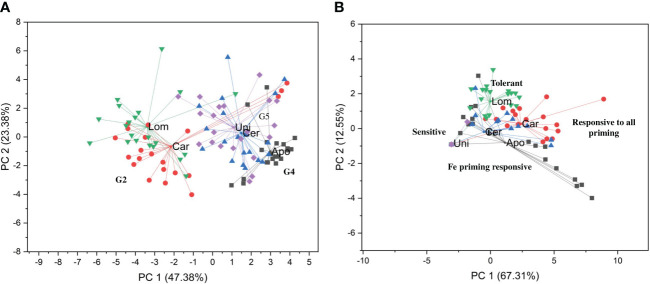
PCA clustering generated using all germination parameters collected from 1 to 10 DAS for the five Italian rice varieties and seed priming treatments. **(A)** PCA plot for optimal germination conditions. **(B)** PCA plot for PEG-induced water stress. Lom, Lomello; Car, Carnaroli; Uni, Unico; Apo, Apollo; Cer, Cerere; G2, G4, G5 genetic clades described by Mongiano et al. (2018) for variety development.

To better evidence the genotype-specific behavior, an integrative analysis was carried out by applying a Principal Component Analysis (PCA) based on all the germination parameters collected from 1 to 10 DAS. Two PCA clusters were generated relative to the data regarding non-stressed (**Figure 4A**) and stressed (**Figure 4B**) conditions. The two principal components (PC) contributed to 70.76% (in which 47.38% belongs to PC1 and 23.38% belongs to PC2) of the observed variability for non-stressed conditions and 79.86% (in which 67.31% belongs to PC1 and 12.55% belongs to PC2) of the variability in case of PEG-induced stress. The clustering pattern obtained from the germination behavior of the seeds grown under optimal condition (**Figure 4A**) grouped the varieties according to the genetic clades described by Mongiano et al. (2018); namely, Carnaroli and Lomello were grouped around G2 clade, Apollo to G4, while Cerere and Unico were grouped to clade G5.

On the other side, the clustering pattern based on the data collected from PEG-induced stress revealed a treatment-based grouping (**Figure 4B**). Apollo and Cerere were grouped closely as responsive to iron treatments (Fe, PGA-Fe, and dPGA-Fe), while Carnaroli clustered separately since this variety was responsive to most seed treatments. Unico, was placed close to Apollo, and Cerere, forming the group of varieties sensitive to PEG-induced stress, as reflected from all the germination data. The difference between Unico and the other sensitive varieties consists in the fact that while Apollo and Cerere were positively affected by priming, Unico was not. Lomello represented another cluster as majority of the germination parameters investigated pointed towards an enhanced tolerance to water deprivation stress. Its position close to Carnaroli is in agreement with the fact that the priming treatments resulted in a further improvement of stress tolerance.

Based on the PCA results, it can be inferred that the G2 clade (including Lomello and Carnaroli) is a potential source of genetic materials that can be used for developing water deprivation stress tolerant varieties. Results from this analysis suggest that the trends in priming responsiveness could reflect the breeding treads presented by Mongiano et al. (2018).

### 3.3 Determination of iron and hydrogen peroxide accumulation during the imposed treatments

Perls Prussian blue staining was performed to measure Fe accumulation in 10 DAS seedlings while DAB staining was used to measure the accumulation of H_2_O_2_.

**Figure 5:**
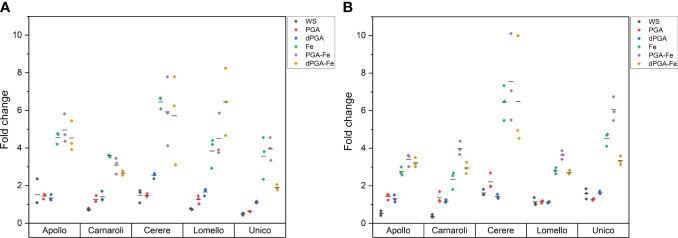
Semiquantitative measurement of iron accumulation through Perls Prussian Blue staining in 10 DAS rice seedlings in the **(A)** absence and **(B)** presence of 25% PEG-induced stress. Horizontal bars indicate the average fold change with respect to the control from the three replicates. PGA, poly-gamma-glutamic acid; dPGA, denatured poly-gamma-glutamic acid; Fe, iron pulsing; WS, water soaking.

Based on the results of Perls Prussian blue staining, it can be observed that, as expected, only the Fe-related priming methods (Fe, PGA-Fe, and dPGA-Fe) displayed Fe accumulation in the germinating seedlings (**Figure 5**). High levels of Fe can be detected in the Cerere variety both in the absence (**Figure 5A**) and presence of PEG (**Figure 5B**). This provides further evidence in support of the previous observation regarding the high response of this variety to priming treatments that includes Fe pulsing (Fe, PGA-Fe, dPGA-Fe). In the absence of stress, most varieties accumulated high Fe levels in the seedlings, except for Carnaroli, which is less responsive to Fe pulsing as evidenced by the phenotyping data. Under PEG-induced stress, Fe levels seem to decrease in most varieties, except Cerere and Unico (**Figure 5B**). This may be due to the fact that Fe requirement is generally enhanced during stress as Fe is needed to supply the energy and redox power necessary to support seedling growth and antioxidant activity (Rout and Sahoo, 2015).

It is well-known that overproduction of ROS can be observed during water stress while stress tolerance is associated with the ability to reduce ROS by increased activity of the antioxidant defense mechanism (Wang et al., 2019). In seeds, accumulation of H_2_O_2_ is also necessary to activate germination since ROS can modulate metabolic and hormone signaling pathways that induce germination (Farooq et al., 2021).

**Figure 6:**
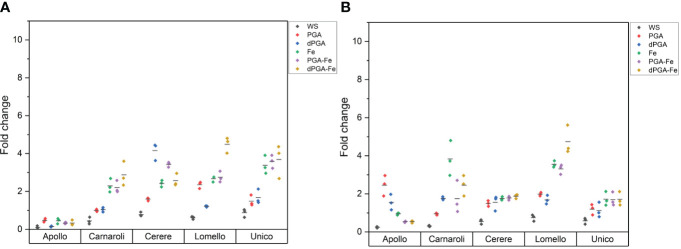
Semiquantitative measurement of H_2_O_2_ staining through DAB staining in 10 DAS rice seedlings in the **(A)** absence and **(B)** presence of 25% PEG-induced stress. Horizontal bars indicate the average fold change with respect to the control from the three replicates. PGA, poly-gamma-glutamic acid; dPGA, denatured poly-gamma-glutamic acid; Fe, iron pulsing; WS, water soaking.

In the case of DAB staining, similar levels of H_2_O_2_ are observed in the presence and absence of stress (**Figure 6**). This may indicate that the proposed priming treatments were effective in balancing ROS production during seedling growth both under optimal and stress conditions. In terms of varieties tested, Apollo showed the lowest observed values in the absence of stress (**Figure 6A**). Considering the priming treatments, dPGA and dPGA-Fe resulted in the highest levels of H_2_O_2_ accumulation in the seedlings of Cerere and Lomello varieties. In the presence of PEG stress (**Figure 6B**), accumulation of H_2_O_2_ was induced by Fe pulsing, specifically for Carnaroli and Lomello varieties. In the case of Apollo, PGA and dPGA also resulted in enhanced H_2_O_2_ accumulation compared to non-stress conditions.

In conclusion, the semiquantitative measurement of Fe and H_2_O_2_ levels in seedlings supported the genotype-specific responses to water deprivation stress and priming treatments.

### 3.4 Changes in gene expression profiles in response to priming and water deprivation stress during the transition from seed to seedlings

To further investigate the observed genotype-specific responses to priming and water stress, qRT-PCR analyses were conducted on genes selected on the basis of the available literature and a preliminary data mining approach (**Supplementary Figure 3**). These genes belong to specific processes like DNA damage response (DDR), antioxidant defense mechanism (ADM), and Fe and Zn homeostasis (Fe/Zn). Additionally, few genes related to drought stress response and amino acid transport (SAAT) were included. DDR genes were selected because DNA damage can accumulate during the early pre-germinative phase, when rapid water uptake generates enhanced ROS production (Rajjou et al., 2012; Pagano et al., 2023). This damage must be repaired prior to cell division to minimize induction of mutations and inhibition of seedling growth and development (Waterworth et al., 2015; Waterworth et al., 2022), therefore DDR genes are envisioned as hallmarks of the seed repair response (Pagano et al., 2019; Forti et al., 2020a; Forti et al., 2020b). Rice genes associated with the antioxidant defense were evaluated as further indicators of stress tolerance (Wang et al., 2019; Panda et al., 2021). Fe/Zn related genes were selected to evaluate metal homeostasis during germination as these are important minerals for metabolic processes during germination. This is in agreement with the fact that the supply of ferrous ions as a priming treatment lead to positive effect on germination, as evidenced by the phenotypic and biochemical analyses.

To address the transition phase from seed to seedlings, the expression of the selected genes was evaluated in Carnaroli and Cerere varieties at different timepoints during germination; namely, in dry seeds (CTRL, 0 h), during seed imbibition (16 h) and at radicle protrusion (3 DAS), both in presence and absence of stress. The two varieties were selected since they exhibited sensitivity to water deprivation stress and responded differently to the imposed priming methods. Specifically, Carnaroli showed efficient responses to all the priming methods while Cerere’s response was mostly related to the presence of iron (Fe, PGA-Fe, dPGA-Fe).

**Figure 7:**
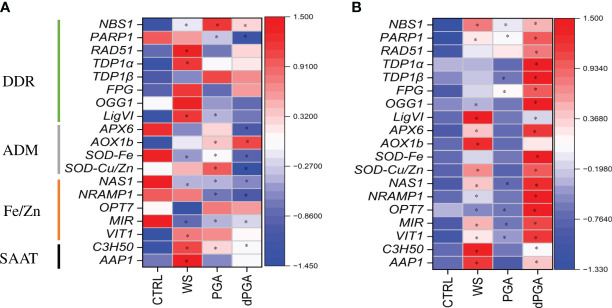
Heatmaps representing the expression patterns of the selected genes during seed imbibition (16 h) in **(A)** Carnaroli and **(B)** Cerere seeds subjected to priming treatments. *p ≤ 0.05 as indicated by the Student *t*-test. CTRL, dry seeds, unprimed control; WS, water soaking; PGA, poly-gamma-glutamic acid; dPGA, denatured poly-gamma-glutamic acid; *LigVI, DNA LIGASE VI; FPG, FORMAMIDOPYRIMIDINE-DNA GLYCOSYLASE; NBS1, NIJMEGEN BREAKAGE SYNDROME 1 MUTATED GENE; OGG1, 8-OXOGUANINE DNA GLYCOSYLASE; OPT7, OLIGOPEPTIDE TRANSPORTER 7; PARP1, POLY [ADP-RIBOSE] POLYMERASE 1; RAD51, RADIATION SENSITIVE PROTEIN 51; TDP1α, TYROSYL-DNA PHOSPHODIESTERASE 1α; TDP1ß, TYROSYL-DNA PHOSPHODIESTERASE 1β; AoX1b, ALTERNATIVE OXIDASE 1B; APX6, ASCORBATE PEROXIDASE 6; Cu/ZnSOD, CU/ZN SUPEROXID DISMUTASE; SODFe, FE SUPEROXID DISMUTASE; MIR, MITOCHONDRIAL-IRON REGULATED; NAS1, NICOTIANAMINE SYNTHASE 1; NRAMP1, NATURAL RESISTANCE-ASSOCIATED PROTEIN 1; VIT1, VACUOLAR IRON TRANSPORTER; AAP1, ASPARTATE AMINOTRANSFERASE 1; C3H50, CYSTEINE 2 HISTIDINE*.

During seed imbibition (16 h), only WS, PGA, and dPGA priming treatments were considered in the absence of stress because this was the timepoint selected for stopping the imposed soaking treatments before proceeding with Fe pulsing and subsequent sowing on water or PEG-containing plates. Control dry, non-primed seeds (CLTR, 0 h) are also included in this comparison. The results are presented as a heatmap (**Figure 7**) where the blue color indicates low expression while red color indicates high gene expression. In the Carnaroli variety, the *PARP1, APX6, SOD-Fe, NAS1, NRAMP1* and *MIR* genes were highly expressed in dry seeds (**Figure 7B**, CTRL). Changes in the expression patterns of the investigated genes were observed in response to the different priming treatments. The WS treatment resulted in a significant upregulation mainly of DDR-related genes (*NBS1, RAD51, TDP1α, LigIV*), and SAAT-related genes (*C3H50, AAP1*). Differently, some of the ADM (*SOD-Fe*) and Fe/Zn-related genes (*MIR*) were downregulated. The PGA priming resulted in significant upregulation of *NBS1*, *AOX1b*, *SOD-Cu/Zn*, and *C3H50* genes and downregulation of *PARP1*, *SOD-Fe*, *NAS1*, *NRAMP1* and *MIR* genes. Further downregulation was observed following dPGA priming where mostly the Fe/Zn homeostasis was involved (*NAS1, NRAMP1, MIR*) along with few DDR (*PARP1*) and ADM (*APX6, SOD-Cu/Zn*) genes. In the Cerere variety, all the tested genes were expressed at low levels in dry seeds (**Figure 7B**, CTRL). In this case, the priming treatments that mostly induced the expression of the genes were dPGA and WS while PGA resulted in a slight upregulation of only few DDR-related genes (*NBS1, PARP1, FPG*). In the case of dPGA, all tested genes were significantly upregulated compared to CTRL, whereas the WS treatment induced the expression of most ADM-, Fe/Zn- and SAAT-related genes.

**Figure 8:**
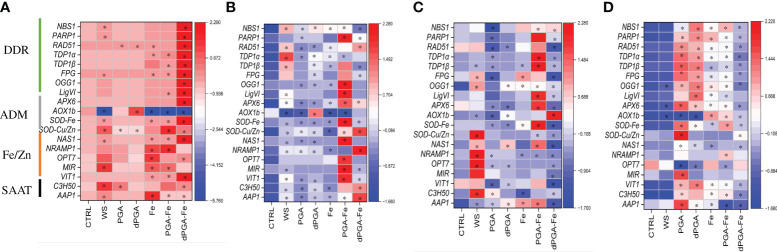
Heatmaps representing the expression patterns of selected genes at 3 days after sowing in presence/absence of priming and PEG stress. **(A)** Carnaroli seedlings under optimal conditions. **(B)** Carnaroli seedlings under PEG-induced stress. **(C)** Cerere seedlings under optimal conditions. **(D)** Cerere seedlings under PEG-induced stress. *p ≤ 0.05 as indicated by the Student *t*-test. CTRL, dry seeds, unprimed control; WS, water soaking; PGA, poly-gamma-glutamic acid; dPGA, denatured poly-gamma-glutamic acid; Fe, iron pulsing; *LigVI, DNA LIGASE VI; FPG, FORMAMIDOPYRIMIDINE-DNA GLYCOSYLASE; NBS1, NIJMEGEN BREAKAGE SYNDROME 1 MUTATED GENE; OGG1, 8-OXOGUANINE DNA GLYCOSYLASE; OPT7, OLIGOPEPTIDE TRANSPORTER 7; PARP1, POLY [ADP-RIBOSE] POLYMERASE 1; RAD51, RADIATION SENSITIVE PROTEIN 51; TDP1α, TYROSYL-DNA PHOSPHODIESTERASE 1α; TDP1ß, TYROSYL-DNA PHOSPHODIESTERASE 1β; AoX1b, ALTERNATIVE OXIDASE 1B; APX6, ASCORBATE PEROXIDASE 6; Cu/ZnSOD, CU/ZN SUPEROXID DISMUTASE; SODFe, FE SUPEROXID DISMUTASE; MIR, MITOCHONDRIAL-IRON REGULATED; NAS1, NICOTIANAMINE SYNTHASE 1; NRAMP1, NATURAL RESISTANCE-ASSOCIATED PROTEIN 1; VIT1, VACUOLAR IRON TRANSPORTER; AAP1, ASPARTATE AMINOTRANSFERASE 1; C3H50, CYSTEINE 2 HISTIDINE*.

At 3 DAS, the gene espression pattern was investigated in response to all priming methods in the presence and absence of PEG-induced stress (**Figure 8**). In this case, the CTRL samples represent seedlings obtained from unprimed seeds. The genotype-specific expression pattern is again quite evident by observing the data obtained under optimal conditions (**Figures 8A, C**) while PEG induced specific changes mosty when considering the different priming treatments (**Figures 8B, C**). In Carnaroli at 3 DAS, all the genes were already highly expressed in the CTRL samples (**Figure 8A**). The priming treatment resulting in a further upregulation of most genes (except for *AOX1b* wich is downregulated) in the dPGA-Fe combination. The Fe and PGA-Fe treatments also resulted in upregulation of Fe/Zn-related genes. Differently, under PEG stress, all the genes were poorly expressed in the CTRL samples for this variety (**Figure 8B**). Significant gene upregulation was mainly observed following PGA-Fe priming while DDR-related genes were induced also by WS. As for Cerere, the genes were expressed at low levels in CTRL samples both in the presence or absence of stress (**Figures 8C, D**). Under optimal conditions, WS mostly induced a significant upregulation of Fe/Zn-related genes while the PGA-Fe priming lead to the upregulation of DDR and ADM genes (**Figure 8C**). Differently, upon PEG stress the priming treatments that generally induce gene upregulation were PGA and dPGA (**Figure 8C**). Gene upregulation was also observed following Fe pulsing (alone or in combination) but at a lower extent.

To investigate if the gene expression trends persisted, 7 DAS seedlings grown under PEG-induced stress were evaluated (**Supplementary Figure 4**). At this timepoint, results were similar in the two varieties. Namely, the CTRL samples were charaterized by high levels of gene expression wherease, in most cases, the priming treatments resulted in downregulation. In Carnaroli, upregulated genes included *NRAMP1* and *OPT7* in response to PGA, and *SOD-Cu/Zn* and *AAP1* in response to Fe and dPGA-Fe. In Cerere, the only upregulated gene was *AOX1b* following dPGA priming.

To summarize, the different germinative behaviour was supported by genotype-specific gene expression patterns following the transition from seeds to seedlings. Specific genes were induced by specific priming treatments both in presence and absence of PEG stress. At 16 h, WS-induced gene upregulation was present in the Carnaroli variety while dPGA mostly affected gene expression in Cerere. PEG treatments added another layer of complexity to the investigated system. In Carnaroli, most genes were downregulated by PEG at 3 DAS in CTRL samples, in agreement with the seeds not being able to germinate under stress in the absence of priming, in contrast with the high expression recorded under optimal conditions. In Cerere, WS stimulates gene expression at 3 DAS under optimal conditions while PGA and dPGA induce the expression of genes under PEG stress. Although it seems difficult to correlate the germination behaviour with the gene expression patters, it can be stated that all genes tested were differentially expressed in a genotype-, priming-, stress- and stage-dependent manner.

## 4 Discussion

In the current study, different seed priming treatments were developed to improve rice germination under water deprivation stress. Given the empirical nature of priming protocols and the influence that the genetic base can have on the efficacy of these treatments, five varieties of high economic impact in the Mediterranean area were tested. In a context where erratic climate changes highly affect crop productivity, screening for tolerant varieties along with the possible applications of easy-to-use, sustainable, and economic treatments to enhance germination and yield is of utmost importance.

The priming protocols developed in this study utilize novel seed priming agents like poly-gamma-glutamic acid (PGA), denatured γ-PGA (dPGA), applied alone or in combination with iron pulsing (Fe). PGA is a biodegradable, non-immunogenic and non-toxic anionic homopolymer made-up of D- and L-glutamic acid units linked by γ-amide bonds (Kimura et al., 2004; Ogunleye et al., 2015). It is mainly produced and secreted by *Bacillus* species (Osera et al., 2009). When released in the environment, γ-PGA contributes to biofilm formation and can help other organisms survive under adverse conditions (Yu et al., 2016). Additionally, the release of glutamic acid subunits from the long polymeric chains can represent a source of carbon and nitrogen (Ogunleye et al., 2015). Since it is biodegradable and non-toxic, γ-PGA has been utilized in a wide array of applications in agriculture, cosmetics, and medical industries. In agriculture, its application generates a positive impact on plant growth, soil microbial community, nitrogen pools, and water/fertilizer use efficiency (Xu et al., 2013; Zhang et al., 2017). Recent reports associated its use to drought tolerance in several plant species, including rapeseed (Xu et al., 2020) and corn (Yin et al., 2018). Currently, there is no report proposing the use of γ-PGA as a seed priming treatment nor has this been tested on rice. Nevertheless, agricultural byproducts, including rice bran, have been used to enhance bacterial PGA production through fermentation (Tang et al., 2015; Song et al., 2019). Moreover, the denatured PGA (formed by single or few glutamic acid residues) can be envisioned as nutripriming, as an exogenous source of glutamic acid. This compound is used by plant cells for the production of GABA (gamma-aminobutyric acid), a key player in the plant response to drought as well as nitrogen mobilizer in germinating seeds (Seifikalhor et al., 2019). Iron pulsing was previously applied to rice seeds by using a ferrous salt (FeSO_4_), and this treatment proved to be favorable during germination (Dey et al., 2021). It also promoted seedling growth by facilitating photosynthesis and nitrogen assimilation, leading to increased chlorophyll content, more efficient activity of the antioxidant system, and ultimately improved yield.

To test whether these priming methods could aid rice seeds germinate better under water deprivation stress, polyethylene glycol (PEG) was used; this agent is widely used to regulate the osmotic potential due to the high hydrophilicity that allows it to sequester water molecules. Previous reports indicated PEG as an ideal system for germination screening due to its high molecular weight that prevents its entry through the plant cell walls while simulating water retention (Islam et al., 2018; Sobahan et al., 2022). According to a 100-rice variety screening study (Islam et al., 2018), as well as other more recent reports (Sobahan et al., 2022), 25% PEG is considered the highest concentration that can be utilized for water stress simulation. Data collected in the present work on multiple parameters related to germination efficiency (G%, GI, MGT, Z), seedling growth (root, shoot, VII) and stress resilience (GDTIMax, SLWTI, RLWTI), evidenced a genotype-specific germination behavior. Carnaroli was responsive to all the priming methods, whereas Cerere and Apollo showed positive changes in the germinative behavior under the treatment with Fe, PGA-Fe, and dPGA-Fe. While the strong genotype-dependent effect of seed priming is an acknowledged feature of these treatments (Harris et al., 2001; Pradhan et al., 2015; Bhardwaj et al., 2021), it also represents a major drawback for designing efficient and universal protocols as it delays the search for common hallmarks of seed quality (Pagano et al., 2023). This was reported also in rice where a recent study investigated the genetic diversity of hydropriming applied under different soil moisture conditions (Nakao et al., 2020). The authors considered 27 genotypes, including *O. sativa* and *O. glaberrima*, representative of either wetland or dryland conditions, and reported wide genotypic differences in priming efficiency with the upland rice showing better responsiveness (Nakao et al., 2020). In our study, the application of integrative PCA further reinforced the genotype-dependent effect while also allowing to attribute each variety to the specific genetic clades that take into consideration the main breeding trends (Mongiano et al., 2018); To this purpose the G2 clade includes Carnaroli and Lomello, G4 includes Apollo, and G5 includes Cerere and Unico. Importantly, under PEG-induced stress, it was possible to also distinguish tolerant (e.g., Lomello) and sensitive (Carnaroli, Cerere, Apollo) or highly sensitive (Unico) varieties. When semiquantitative measurement of Fe and H_2_O_2_ levels were carried out in seedlings, the genotype-specific responses to water stress and priming treatments were further reinforced. The fact that H_2_O_2_ has not accumulated in stressed seedlings when priming was applied further supports the protective effect of the proposed treatments. This is in agreement with other studies carried out in rice showing that different priming techniques (hydropriming, application of selenium or salicylic acid) were able to lower ROS production under heavy metal pollution and nutrient starvation, leading to improved germination (Khan et al., 2020).

**Figure 9:**
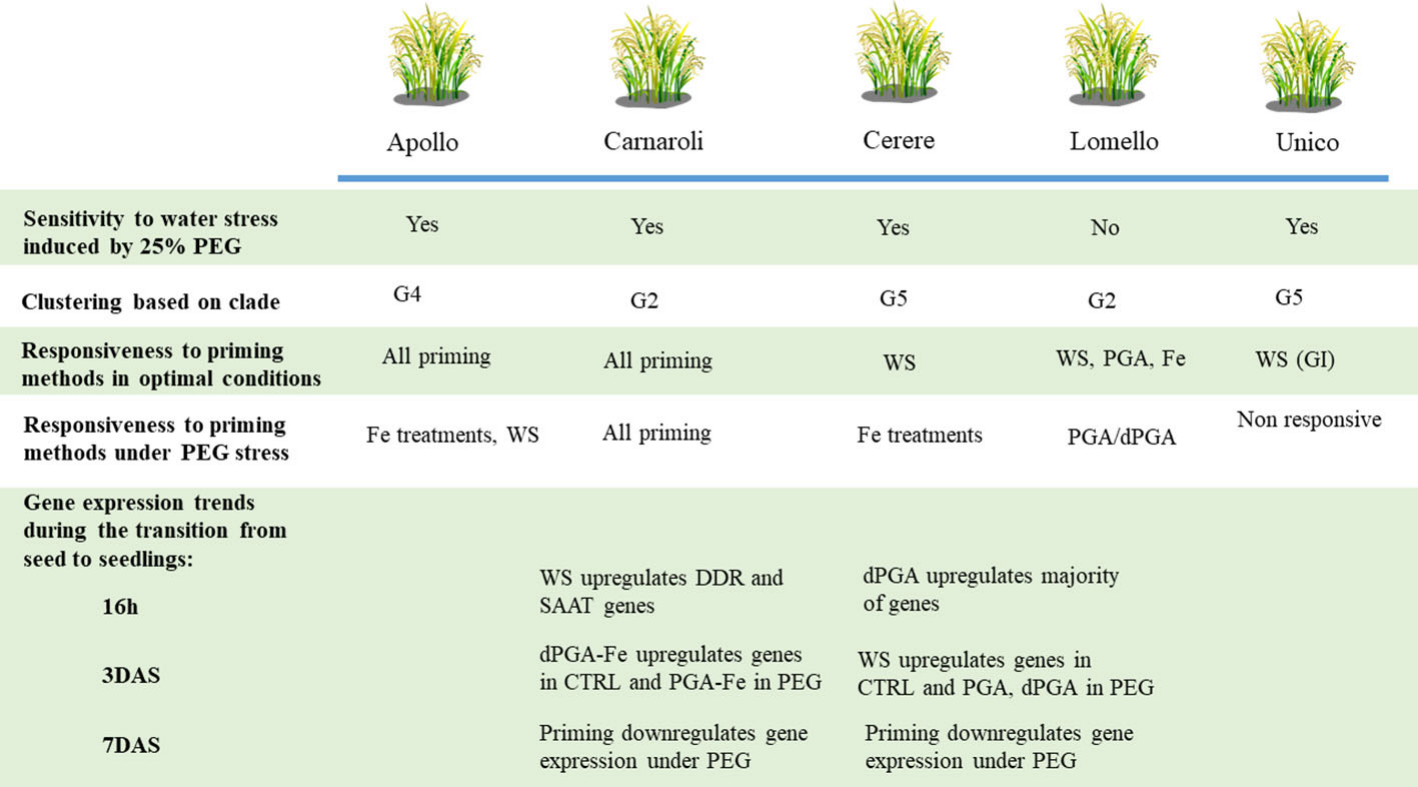
Overview of the main results obtained in the current study. The sensitivity/tolerance to water deprivation stress and responsiveness to priming treatments is considered for each investigated variety. Varietal clustering based on genetic clades is provided. Gene expression trends during the transition from seed to seedling are provided for the Carnaroli and Cerere varieties.

A schematic representation of the response to PEG-induces stress and the effect of seed priming treatments developed in this study is provided in **Figure 9**, taking into consideration the phenotyping and molecular analyses. The data pinpoints the clade distinction between the five popular rice Italian varieties along with their sensitivity/tolerance to water deprivation stress, and their responsiveness to priming. Differences observed among the varieties may be related to their seed type and breeding programs. Carnaroli, a variety obtained through hybridization and part of the G2 clade, is characterized by long grains. Cerere, a variety obtained through mutagenesis and part of the G5 clade, is characterized by the presence of short oval grains (Mongiano et al., 2018). Our study shows that both varieties are sensitive to PEG-induced stress while enhanced tolerance is gained after priming, possibly because it differentially modulates the expression of genes involved in the seed stress response.

To further explore the genotype-dependent response to priming, a targeted gene expression profile study was carried out on Carnaroli and Cerere varieties particularly focusing on the transition from seed to seedlings. A panel of genes was investigated to cover processes related to DDR, antioxidant defense, Fe/Zn homeostasis and stress response. Among the genes associated with DDR, *NBS1* (Nijmegen breakage syndrome 1 mutated gene), is a component of the MRN (MRE11-RAD50-NBS1) double-strand breaks sensor complex, while *RAD51* is directly involved in HR (homologous recombination) (Banerjee and Roy, 2021). Additional genes involved in BER (Base Excision Repair) were also selected as this type of repair pathway is often associated with damage induced by accumulation of ROS (Roldán-Arjona and Ariza, 2009). The *FPG* (formamidopyrimidine-DNA glycosylase) gene, encoding for a DNA repair enzyme that excises oxidized purines from damaged DNA, and the *OGG1* (8-oxoguanine glycosylase) gene, involved in the removal of 8-oxoG products, were proven to be highly induced during seed imbibition in multiple species (Macovei et al., 2011; Forti et al., 2021; Kowalik and Groszyk, 2023). The two *TDP1* (tyrosyl-DNA phosphodiesterase 1) genes, *TDP1α* and *TDP1β*, encoding enzymes involved in hydrolyzing phosphodiester bond formed between topoisomerase I and the 3’-DNA, were proved to be highly active during seed imbibition as well as seedling growth under stress (Macovei et al., 2010; Gualtieri et al., 2021). *PARP1* (poly-(ADP-ribose) polymerase 1) gene plays an important role in mediating the response to DNA damage during gametophyte development (Banerjee and Roy, 2021). The *LigVI* (DNA ligase VI) gene is a plant-specific ligase essential for DNA ligase-mediated rejoining of single- and double-strand breaks, previously identified as a major determinant of seed quality in *A. thaliana* (Waterworth et al., 2010). Antioxidant-related genes, such as *APX6*, encoding for an ascorbate peroxidase isoform found in the mitochondria which participates in H_2_O_2_ detoxification, was shown to have a more stable expression compared with other isoforms (Rossatto et al., 2017). The *AOX1b* gene encodes an alternative oxidase known to be induced by several stresses, including oxidative stress caused by H_2_O_2_ exposure (Feng et al., 2009). The superoxide dismutases *SOD-Fe* and *SOD-Cu/Zn* were proven to be highly expressed in rice during salt and/or osmotic stress (Rosatto et al., 2017). For Fe/Zn genes, *NAS1* (nicotianamine synthase 1) is the gene related to Fe and Zn uptake and transport and it is highly expressed during the early stage of germination in rice (Nozoye et al., 2007). The *NRAMP1* (natural resistance-associated macrophage protein 1) gene is also associated with metal transport, being part of an important protein transporter family, highly expressed during germination (Nozoye et al., 2007). The *OPT7* (iron deficiency-regulated oligopeptide transporter 7) gene is associated with transport of ferrous ions bound to nicotianamine (Bashir et al., 2015). Another metal transporter coding gene is *VIT1* (vacuolar iron transporter), associated with Fe accumulation and reallocation during seed development and germination (Eroglu et al., 2017). Among other stress-related genes, *C3H50* (Cystenin3-Histidine50) encodes a zinc finger protein, highly induced by drought in rice via post-transcription regulation of messenger RNAs (Seong et al., 2020). This gene showed the highest expression among various C3H orthologs, especially in roots during drought stress, maybe because it has the highest number of drought-responsive elements (DRE). The *AAP1* (amino acid transporter protein 1) gene, encoding for a member of the AAT protein family, was shown to absorb and translocate a wide variety of amino acids (Ji et al., 2020). Therefore, the genes selected for this investigation cover broad aspects of the seed response to stress both during imbibition and seedling establishment. The data collected in this study indicate that these genes are differentially expressed in Carnaroli and Cerere both from the point of view of the temporal transition as well as considering the priming methods and stress treatment (**Figure 9**). When considering seed imbibition, the WS treatment mostly resulted in gene upregulation in Carnaroli, while in Cerere dPGA had the same effect. At the radicle protrusion stage (3 DAS), further differences were evidenced among the two varieties, as the investigated genes are highly expressed in Carnaroli while low expression values were obtained for Cerere under control conditions. Under PEG-induced stress, PGA-Fe and dPGA-Fe treatments had a greater influence on the gene expression in Carnaroli, while in Cerere mostly PGA and dPGA priming affected the expression of some genes. However, these differences subsided at later seedling development (7 DAS), when the majority of genes were downregulated by priming. This may indicate that, at a molecular level, the priming effect is fundamental during the early phases of gemination to boost seed germination and stress response.

Given the results obtained in this study, the proposed seed priming treatments can be envisioned as sustainable and versatile practices to help addressing the impact of water deprivation stress in rice. Considering the observed genotype-specific differences, further varieties should be screened and the priming treatments can be tested also in relation to other types of stress. Ultimately, filed trials should be envisioned to ascertain the effect of these priming methods on final yield under drought conditions.

## Data availability statement

The original contributions presented in the study are included in the article/**Supplementary Material**. Further inquiries can be directed to the corresponding author.

## Author contributions

CD: Data curation, Formal analysis, Investigation, Methodology, Validation, Writing – review & editing. AP: Data curation, Formal analysis, Validation, Writing – review & editing. CC: Formal analysis, Supervision, Visualization, Writing – review & editing. DS: Formal analysis, Methodology, Writing – review & editing. IS-L: Funding acquisition, Investigation, Resources, Visualization, Writing – review & editing. AB: Resources, Validation, Writing – review & editing. AM: Conceptualization, Data curation, Investigation, Supervision, Writing – original draft.
