# Supplementary material for: Genotype-specific germination behavior induced by sustainable priming techniques in response to water deprivation stress in rice
Source: Front Plant Sci. 2024 Feb 8;15:1344383. doi: 10.3389/fpls.2024.1344383 (PMC10881859; doi:10.3389/fpls.2024.1344383)
Supplement: Supplementary file 1 [file DataSheet_1.docx]

Supplementary Material

Genotype-Specific Germination Behavior Induced by Sustainable Priming Techniques in Response to Water Deprivation Stress in Rice

Conrado Dueñas, Jr.^1^, Andrea Pagano^1^, Cinzia Calvio^1^, Dhanush Srikanth Srikanthan^1^, Inez Slamet-Loedin^2^, Alma Balestrazzi^1^, Anca Macovei^1,*^

^1^ Department of Biology and Biotechnology ‘L. Spallanzani’, University of Pavia, Pavia, Italy

^2^ Trait and Genome Engineering Cluster, Rice Breeding Innovations, International Rice Research Institute, DAPO Box 7777, Metro Manila 1277, Philippines

*** Correspondence:** Corresponding Author: [anca.macovei@unipv.it](mailto:anca.macovei@unipv.it)

**Supplementary Table 1.** Abbreviations, units, and methodology to determine germination indices, seedling traits, and drought tolerance indices.

| Parameter | Unit | Formula of calculation | Description of formula | |
| --- | --- | --- | --- | --- |
| Germination Indices | |  |  | |
| G% | % | G%=(no of seeds germinated / to number sown) x 100 |  | |
| GI | - | GI=(10×n1) + (9×n2) + · · · + (1×n10) | n1, n2… n10 = No. of germinated seeds on the first and subsequent days until the last day; 10, 9 … and 1 are weights given to the number of germinated seeds | |
| MGT | day | MGT=∑ f ·x/ ∑ f | f= seed germinated on day x | |
| Z | - | Z = ∑C_ni_, _2_ / N | Where ∑C_ni, 2_=∑(ni(ni-1)/2) while N= ∑ni( ∑ni-1)/2 | |
| Seedling Trait |  |  |  | |
| Root | mm | measured using ImageJ program |  | |
| Shoot | mm | measured using ImageJ program |  | |
| VII | - | VII = (Root +Shoot) x G% at 7DAS |  | |
|  |  |  |  | |
| Water Stress Tolerance Indices | | |  | |
| GWTIMax | % | (G% at 7DAS under stress conditions/G% at 7DAS under non stressed condition) * 100 |  |  |
| SLWTI | % | (StLn under stress conditions/StLn under non-stressed condition) * 100 |  | |
| RLWTI | % | (RtLn under stress conditions/RtLn under non-stressed condition) * 100 |  | |

**Supplementary Table 2**. List of oligonucleotide sequences used for the qRT-PCR reactions. Genebank accession numbers are provided for each gene.

| Target gene | Accession no. | Forward sequence (5'-3') | Reverse sequence (5'-3') |
| --- | --- | --- | --- |
| *LigVI* | AK064463 | CAACCACCCCTCTCTGATGG | GCAAGCAGCGTGTAATTGCT |
| *FPG* | AK065376 | GATCATCAAGGACCGCATCG | TGGTACAGTAGCGCACAAGA |
| *NBS1* | AK069561 | ACTGGCAATGCAACCTTCAG | CCAAAACCGCATCTAGGAGC |
| *OGG1* | CI144528 | AGTTCCTCTGCTCCTCCAAC | CGACGATGTACTTTGCCCTG |
| *OPT7* | AK102404 | CGTCATCATCACCATCTTCG | GCCCTGAAGAGTGAGACCTG |
| *PARP1* | AK103479 | GAGATCCCTGATGCTCGTCG | TGCTCTTCATTGTCGGCTGT |
| *RAD51* | AB080264 | TGCGAGCCAACTTCAT | AGAGCCAGTTTCTATCCC |
| *TDP1α* | AK069279 | TCGGTGTCAAGCATTCCTATC | CCTTCCACTACCGCTTCAAT |
| *TDP1ß* | AK120071 | TCGGTGTCAAGCATTCCTATC | CCTTCCACTACCGCTTCAAT |
| *AoX1b* | AB004865 | GATGCCTGCTCAGTTCATCA | GGCATAAAACGGAGTGACAA |
| *APX6* | AK061107 | GAAGCTGATCCAGCCCATCA | CGGCTTCCTCAATGGCTGT |
| *CuZnSOD* | AK243377 | GGTGTTGCTAATGTCAATGTCTC | ATCATCAGGATCAGCATGGAC |
| *SODFe* | AK111656 | CAAGTCACAAACCCAGAGTCAT | GGAATACAAGATGTCAGGCTCA |
| *MIR* | AK103636 | CGTCATGGTCTTCGGTCTCCTACGTGCTCG | GCTAGTCGTTGTCAACAGTCAACACAAAGA |
| *NAS1* | AK112069 | GTCTAACAGCCGGACGATCGAAAGG | TTTCTCACTGTCATACACAGATGGC |
| *NRAMP1* | AK103557 | CGGTGTTGGCTGGTTTTTAT | CATTCTGCCAATCTGCCAAT |
| *Vit1* | AK071589 | TCCCTCTCCTTCCCTACATG | TTCCAGTAAAGCGACCCTTG |
| *AAP1* | AK106110 | GCACATTACAAGCCATTCAGCGTC | CTGACGAAACACTTGAGCACTC |
| *C3H50* | AJ575241 | TATCGCACAAGGCTCTGCAA | CAATGGATGAAGAAGCGCGG |
| *25S rNA* | AK119809 | AAGGCCGAAGAGGAGAAAGGT | CGTCCCTTAGGATCGGCTTAC |

**Supplementary Table 3**. Germination parameters calculated under non-stressed conditions. Gmax, germination percentage at 10 days after sowing; GI, germination index; MGT, mean germination time; Z, synchronicity; VII, vigor index; Root, root length; Shoot, shoot length. Samples followed by different letters indicate statistically significant (*p* ≤ 0.05) differences determined using the ANOVA Tukey’s test.


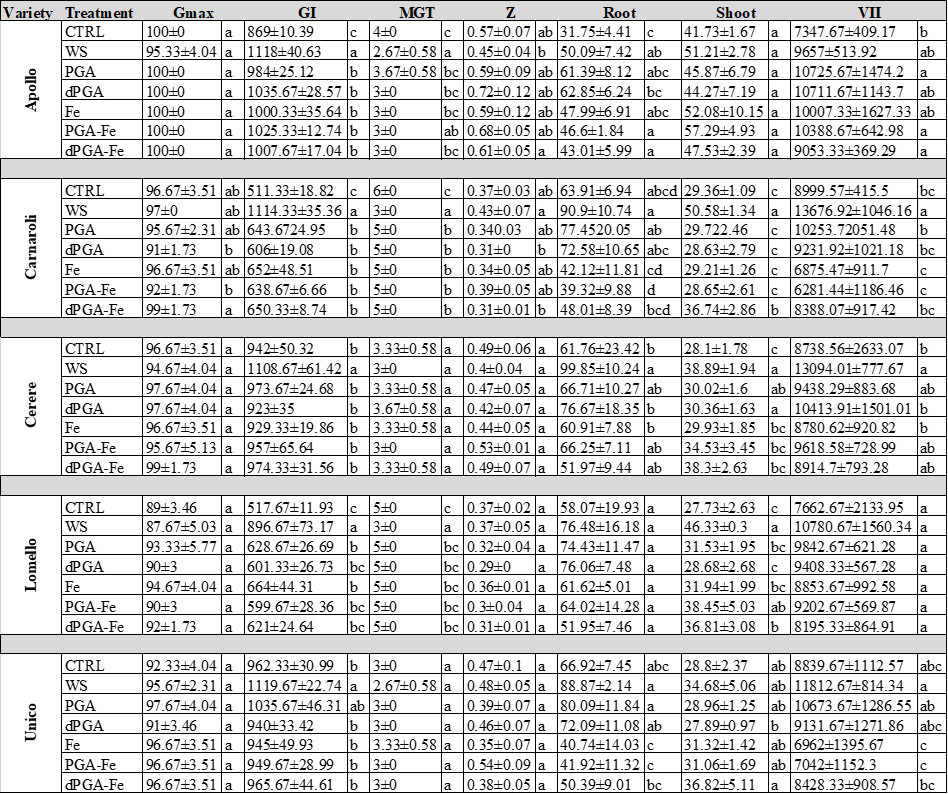


**Supplementary Table 4**. Germination parameters calculated in the presence of 25% PEG-induced water stress. Gmax, germination percentage at 10 days after sowing; GI, germination index; MGT, mean germination time; Z, synchronicity; VII, vigor index; Root, root length; Shoot, shoot length. Samples followed by different letters indicate statistically significant (*p* ≤ 0.05) differences determined using the ANOVA Tukey’s test.


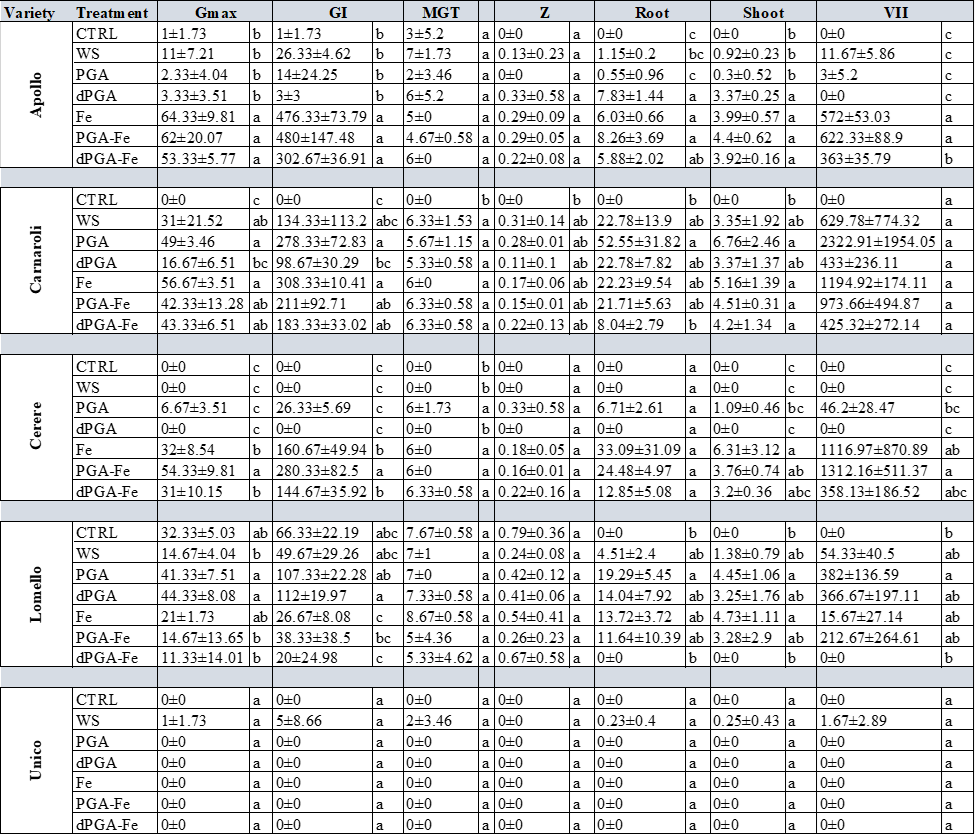


**Supplementary Table 5**. Statistical analysis presented for GWTI data calculated daily for 10 days (GWTI1 – GWTI10) for the five Italian rice varieties in response to priming and PEG-induced stress. Samples followed by different letters indicate statistically significant (*p* ≤ 0.05) differences determined using the ANOVA Tukey’s test.

**
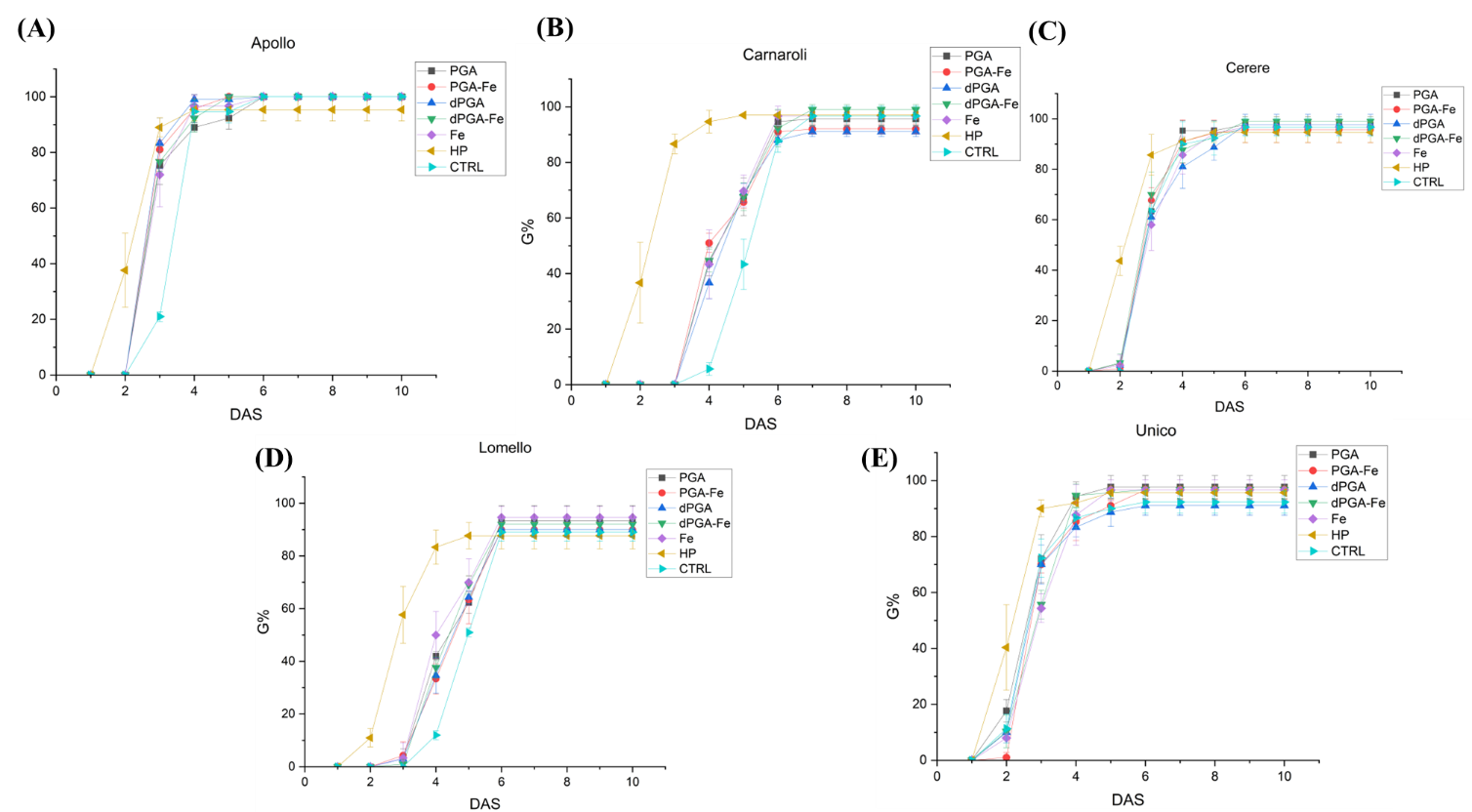
**

**Supplementary Figure 1.** Germination percentage (%) of **(A)** Apollo, **(B)** Carnaroli, **(C)** Cerere, **(D)** Lomello, and **(E)** Unico varieties under non-stressed conditions. CTRL, unprimed control; PGA, poly-gamma-glutamic acid; dPGA, denatured poly-gamma-glutamic acid; Fe, iron pulsing; WS, water soaking; DAS, days after sowing.

**
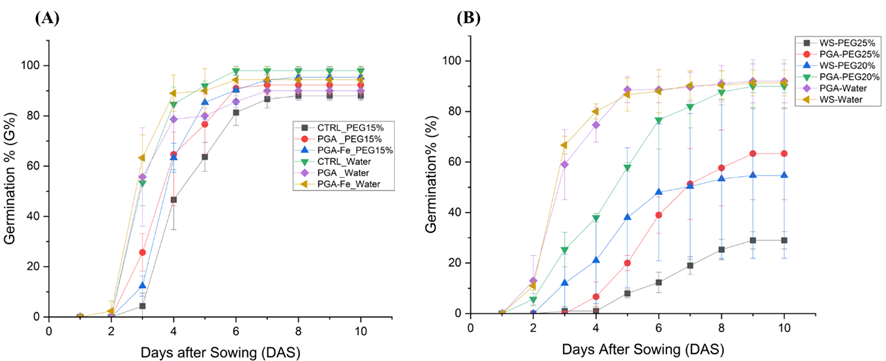
**

**
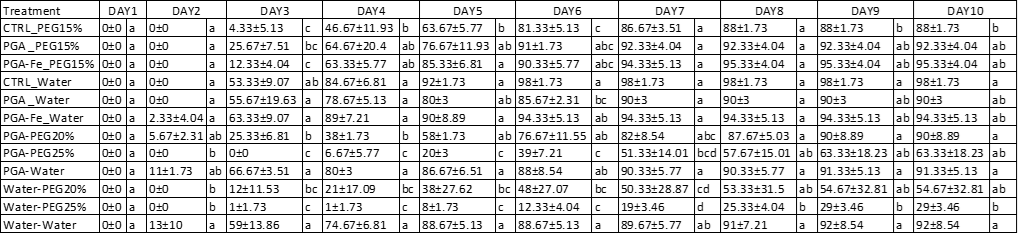
**

**Supplementary Figure 2.** Germination percentage (%) of Lomello seeds treated with PGA, dPGA, and WS and sown in **(A)** 15% PEG or **(B)** 20% and 25% PEG concentrations along with no stress water controls. The germination was monitored daily for 10 days (DAS, days after sowing). Statistical significance (*p* ≤ 0.05) as revealed by Tukey’s test is given below the figures.

**
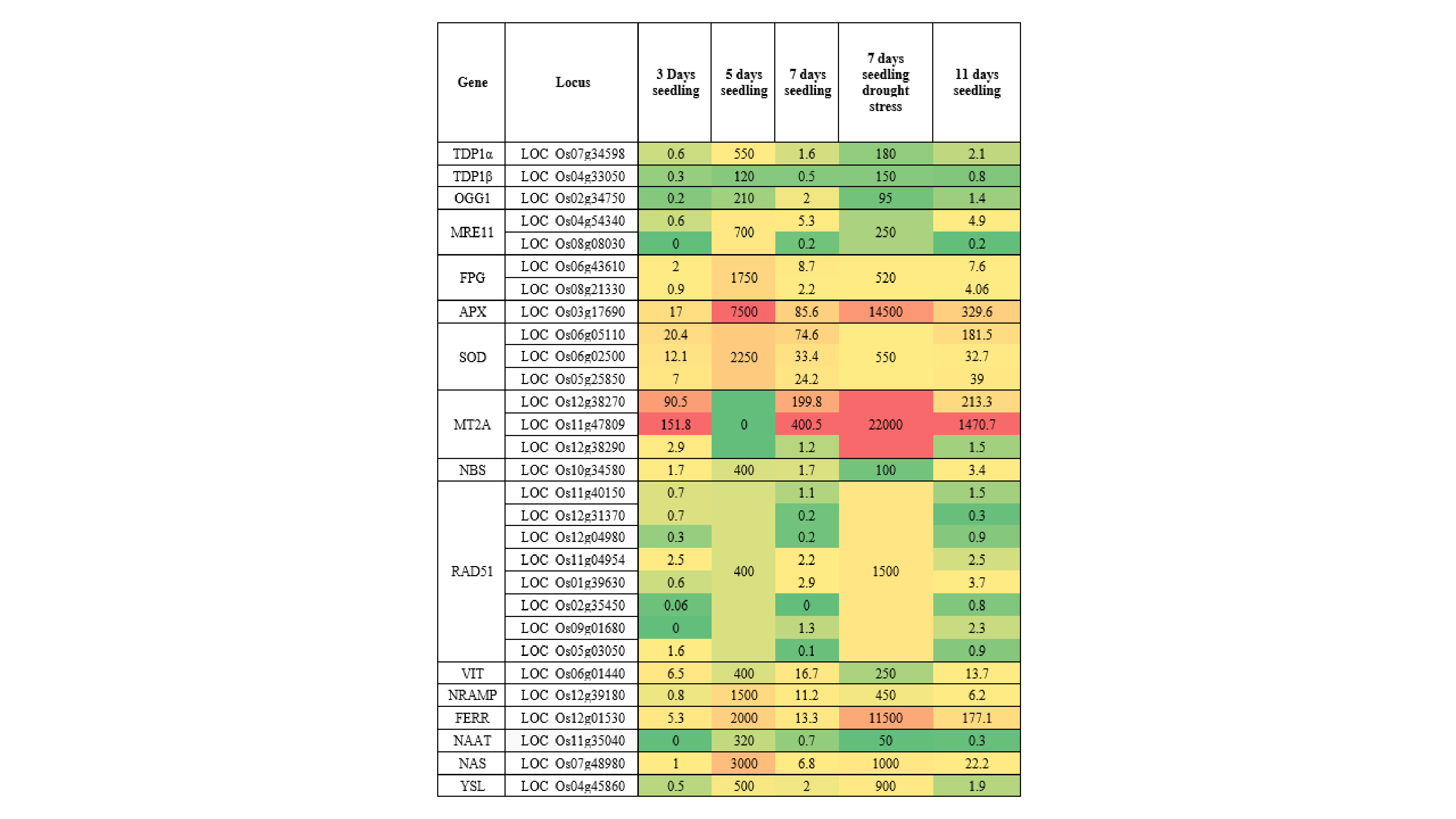
**

**Supplementary Figure 3**. Gene expression data mining approach to evaluate the expression of genes involved in DNA Damage Response (DDR), antioxidant response, and iron uptake genes in several rice databases. Rice Genome Annotation Project (<http://rice.uga.edu/>) was used to obtain genes and isoforms annotations. Rice Expression Database (RED, <http://expression.ic4r.org/>), PlaNet (<http://aranet.mpimp-golm.mpg.de/index.html>) and Rice Expression Profile Database (RiceXpro, <https://ricexpro.dna.affrc.go.jp/>) databases were used to extract the gene expression values from quality-controlled RNA-seq and microarray data encompassing diverse tissues and treatments. The current data mining set was designated to focus on specific tissues and stages related to germination namely, 3 days seedling, 5 days seedling, 7 days seedling, 7 days seedling under drought stress and 11 days seedling. The list of analyzed genes included:

- DDR-related genes: *TDP1α* (LOC_Os07g34598), *TDP1β* (LOC_Os04g33050), *OGG1* (LOC_Os02g34750), *MRE11* (LOC_Os04g54340, LOC_Os08g08030), *FPG* (LOC_Os06g43610, LOC_Os08g21330), *NBS1* (LOC_Os10g34580), *RAD*s (LOC_Os11g40150, LOC_Os12g31370, LOC_Os12g04980, LOC_Os11g04954, LOC_Os01g39630, LOC_Os02g35450, LOC_Os09g01680, LOC_Os05g03050).

- Antioxidant response-related genes: *APX* (LOC_Os03g17690), *SOD* (LOC_Os06g05110, LOC_Os06g02500, LOC_Os05g25850), *MT2A* (LOC_Os12g38270, LOC_Os11g47809, LOC_Os12g38290)

- Iron homeostasis-related genes: *VIT* (LOC_Os06g01440), *NRAMP* (LOC_Os12g39180), FERR (LOC_Os12g01530), *NAAT* (LOC_Os11g35040), *NAS* (LOC_Os07g48980), *YSL* (LOC_Os04g45860).

Highly expressed genes in 5-days old seedlings included *FPG, APX, SOD, NRAMP, FERR, NAS*. Highly expressed genes in 7-days old seedlings included *MT2A*, and *APX*. Highly expressed genes under drought included *APX, MT2A*, and *FERR*. These genes may represent potential hallmarks to distinguish between different germination behavior under normal growth or stress conditions.

Single, most-expressed isoforms were further selected to conduct qRT-PCR analysis during seed germination in the proposed experimental system.


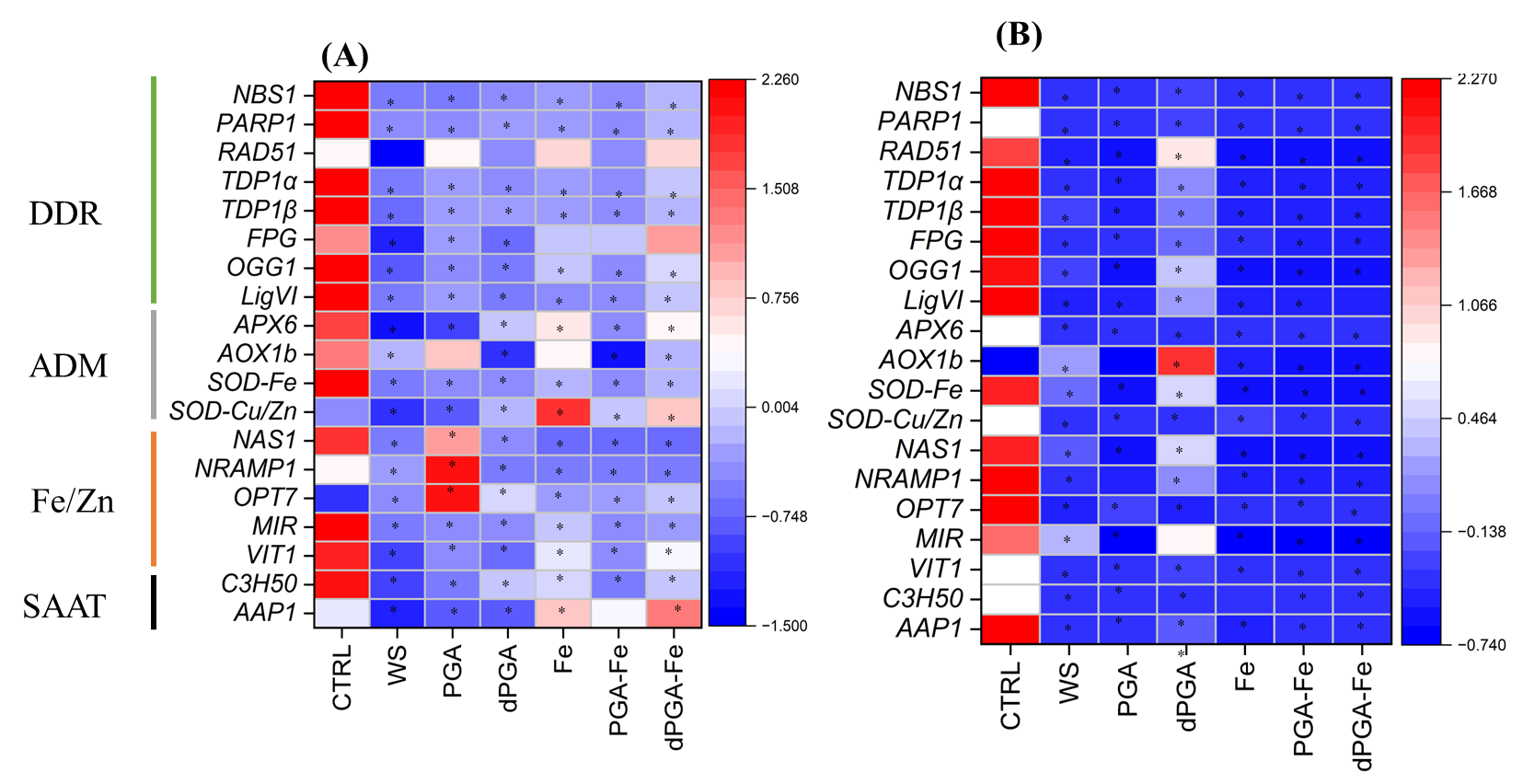


**Supplementary Figure 4**. Heatmaps representing the expression patterns of selected genes at 7DAS under PEG-induced stress in **(A)** Carnaroli and **(B)** Cerere seeds subjected to priming treatments. CTRL, dry seeds, unprimed control; WS, water soaking; PGA, poly-gamma-glutamic acid; dPGA, denatured poly-gamma-glutamic acid; *NBS1*, Nijmegen Breakage Syndrome 1; *PARP1*, Poly ADP-ribose polymerase-1; *TDP1α*, Tyrosil-DNA Phosphodiesterase 1-alpha; *TDP1β*, Tyrosil-DNA Phosphodiesterase 1-beta; *FPG*, formidopyrimidine DNA glycosylase; *OGG1*, 8-oxoguanine DNA glycosylase-1; *APX6*, ascorbate peroxidase 6; *AoX1b*, Alternate oxidase 1b; *SOD*, superoxide dismutase; *NAS1*, nicotinamine synthase 1; *NRAMP1*, natural resistance-associated macrophage protein 1; *OPT7*, oligopeptide transporter 7; *MIR*, mitochondrial iron-regulated; *VIT1*, vacuolar iron transporter 1; *C3H50*, cystenin 2 histidine; *AAP1*, aspartate aminotransferase-1. *, *p* ≤ 0.05 as indicated by the Student *t*-test.
